# Examining Variation in Life Expectancy Estimates by ZIP Code Tabulation Area (ZCTA) in Hawaii’s Four Main Counties, 2008–2012

**DOI:** 10.5888/pcd15.180035

**Published:** 2018-09-20

**Authors:** Joshua R. Holmes, Joshua L. Tootoo, E. Julia Chosy, Amber Y. Bowie, Ranjani R. Starr

**Affiliations:** 1 Surveillance, Evaluation, and Epidemiology Office, Chronic Disease Prevention and Health Promotion Division, Hawai`i State Department of Health, Honolulu, Hawai`i; 2 Children’s Environmental Health Initiative, Rice University, Houston, Texas; 3 Hawai`i Health Data Warehouse, Research Corporation of the University of Hawaii, University of Hawai`i, Honolulu, Hawai`i

**Figure:**
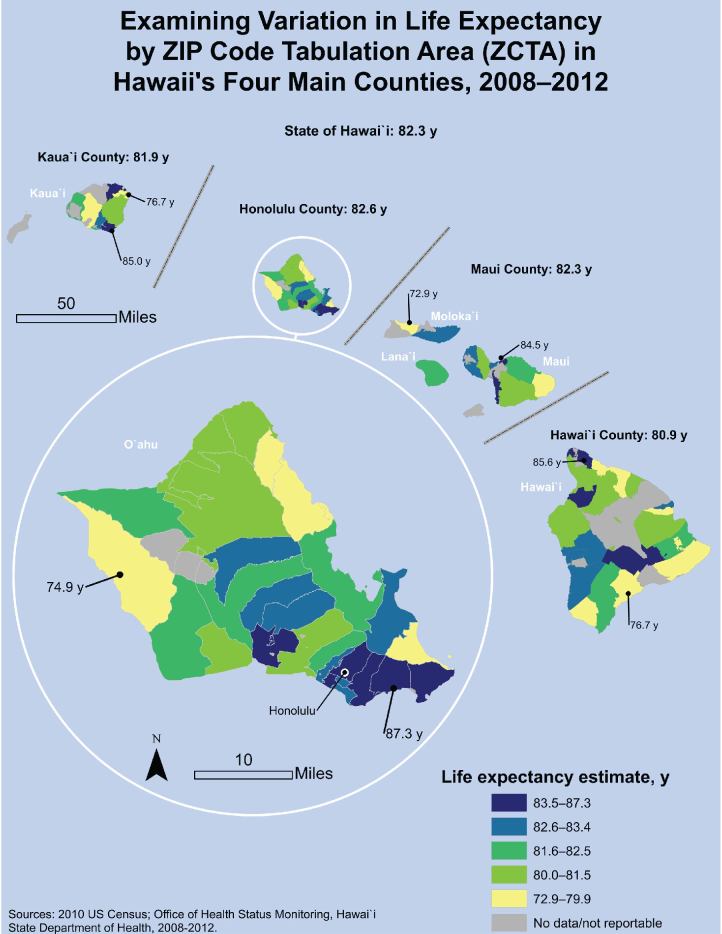
Despite comparable county-level estimates in Hawai`i, substantial variations in life expectancy exist by ZIP Code Tabulation Area (ZCTA) (14.4 years between the highest life expectancy and the lowest life expectancy), highlighting the importance of examining data at small geographic scales to identify spatial health disparities. The map helps enhance awareness of regions of high need for targeted funding allocation and public health interventions. Life expectancy estimates were grouped into quintiles; for each county, the ZCTA with the lowest estimate and the ZCTA with the highest estimate are indicated. **Table:** 

Area Name	Life Expectancy Estimate, y	95% Confidence Interval
Hawai'i State	82.3	82.1–82.4
Honolulu County	82.6	82.4–82.7
ZCTA 96821	87.3	86.3–88.3
ZCTA 96814	85.6	84.3–86.9
ZCTA 96825	85.6	84.9–86.3
ZCTA 96822	85.2	84.5–85.9
ZCTA 96860	85.0	84.1–85.8
ZCTA 96816	83.8	83.2–84.5
ZCTA 96818	83.4	82.6–84.2
ZCTA 96734	83.4	82.8–84.0
ZCTA 96782	83.4	82.6–84.1
ZCTA 96826	83.2	82.4–84.0
ZCTA 96815	83.2	82.2–84.1
ZCTA 96701	83.0	82.3–83.7
ZCTA 96789	83.0	82.3–83.6
ZCTA 96813	82.9	81.9–83.8
ZCTA 96817	82.5	81.8–83.2
ZCTA 96850	82.3	82.3–82.3
ZCTA 96791	82.0	80.3–83.8
ZCTA 96797	82.0	81.4–82.6
ZCTA 96707	81.8	80.9–82.8
ZCTA 96744	81.5	81.0–82.1
ZCTA 96857	81.3	77.4–85.2
ZCTA 96819	81.2	80.5–81.9
ZCTA 96706	81.0	80.4–81.7
ZCTA 96786	80.6	79.8–81.5
ZCTA 96731	80.3	77.0–83.6
ZCTA 96712	80.1	78.3–81.9
ZCTA 96717	79.9	77.5–82.4
ZCTA 96762	79.9	77.2–82.5
ZCTA 96730	76.8	72.9–80.8
ZCTA 96795	76.0	74.4–77.5
ZCTA 96792	74.9	74.2–75.7
ZCTA 96759	No data/not reportable	
ZCTA 96853	No data/not reportable	
ZCTA 96859	No data/not reportable	
ZCTA 96863	No data/not reportable	
Maui County	82.3	81.8–82.7
ZCTA 96779	84.5	81.0–87.9
ZCTA 96753	83.8	82.7–84.9
ZCTA 96761	83.1	81.7–84.4
ZCTA 96732	82.7	81.6–83.7
ZCTA 96748	82.6	79.5–85.7
ZCTA 96763	82.5	80.2–84.8
ZCTA 96708	82.2	80.5–83.9
ZCTA 96768	81.8	80.6–83.0
ZCTA 96793	81.0	80.1–81.9
ZCTA 96790	80.7	79.1–82.3
ZCTA 96713	77.4	74.2–80.6
ZCTA 96729	72.9	69.4–76.3
ZCTA 96742	No data/not reportable	
ZCTA 96757	No data/not reportable	
ZCTA 96770	No data/not reportable	
Kaua`i County	81.9	81.3–82.5
ZCTA 96756	85.0	82.6–87.4
ZCTA 96754	84.2	81.3–87.0
ZCTA 96741	83.2	80.8–85.6
ZCTA 96796	82.5	79.1–86.0
ZCTA 96705	82.4	79.8–85.0
ZCTA 96752	82.2	79.4–85.1
ZCTA 96746	81.2	80.2–82.3
ZCTA 96766	81.1	79.9–82.4
ZCTA 96716	79.6	76.6–82.6
ZCTA 96703	76.7	73.6–79.7
ZCTA 96714	No data/not reportable	
ZCTA 96722	No data/not reportable	
ZCTA 96747	No data/not reportable	
ZCTA 96751	No data/not reportable	
ZCTA 96765	No data/not reportable	
ZCTA 96769	No data/not reportable	
Hawai'i County	80.9	80.5–81.3
ZCTA 96755	85.6	82.7–88.5
ZCTA 96785	85.1	82.2–88.1
ZCTA 96738	84.6	81.9–87.4
ZCTA 96750	82.9	79.8–86.0
ZCTA 96704	82.7	80.2–85.1
ZCTA 96783	82.6	79.5–85.8
ZCTA 96772	82.5	78.8–86.2
ZCTA 96749	82.0	80.6–83.4
ZCTA 96771	81.5	79.5–83.6
ZCTA 96776	81.5	78.3–84.7
ZCTA 96740	81.3	80.4–82.2
ZCTA 96725	81.0	78.3–83.8
ZCTA 96743	80.7	79.2–82.3
ZCTA 96720	80.3	79.7–81.0
ZCTA 96781	79.9	76.9–82.8
ZCTA 96778	79.5	78.0–81.0
ZCTA 96760	78.6	75.6–81.5
ZCTA 96727	78.4	76.5–80.4
ZCTA 96737	77.5	74.5–80.5
ZCTA 96777	76.7	72.9–80.4
ZCTA 96710	No data/not reportable	
ZCTA 96719	No data/not reportable	
ZCTA 96726	No data/not reportable	
ZCTA 96728	No data/not reportable	
ZCTA 96764	No data/not reportable	
ZCTA 96773	No data/not reportable	
ZCTA 96774	No data/not reportable	
ZCTA 96780	No data/not reportable

## Background

Evidence shows that a person’s ZIP Code can affect his or her health. Health disparities are often geographically concentrated. For example, areas that lack sidewalks and safe places to exercise have lower levels of physical activity and higher rates of obesity than areas that have these features. Neighborhoods with poor access to fresh fruits and vegetables and a high density of fast food restaurants have higher rates of obesity than neighborhoods with good access and a low density. Areas with poor access to primary health care, compared with areas with good access, have lower levels of use of preventive health services and a higher burden of chronic disease. Geographic areas with a constellation of risk factors, including those related to the social determinants of health, can lead to disproportionately poor health outcomes (1). Visualizing the distribution of health outcomes at small geographic scales, such as neighborhoods and ZIP codes, is therefore critical; using subcounty geographic units helps identify neighborhoods with the greatest need for intervention.

Life expectancy estimates are often used to compare population health across geographic regions because life expectancy is understood by the public, has well-established methodologies, and is influenced by many factors. Historically, Hawai`i has had the highest life expectancy of any state (2). By county, Honolulu County has the highest life expectancy in Hawai`i (3); to date, no subcounty life expectancy estimates for Hawai`i have been published. We aimed to elucidate variation in life expectancy by ZIP Code Tabulation Area (ZCTA) across Hawai`i.

## Methods

Hawai`i comprises 5 counties, with a total population of 1,360,301 in 2010. More than 950,000 reside in Honolulu County, which includes the island of O`ahu and has the highest population density (8). Hawai`i County, which consists of the island of Hawai`i, has more than 185,000 residents and the lowest population density (8). Maui County (including Kalawao County) comprises the islands of Lana`i, Maui, Moloka`i and Kaho`olawe (uninhabited), with a total population of almost 155,000 residents. Finally, the least populated county, Kaua`i County, consists of the islands of Kaua`i and Ni`ihau, with approximately 67,000 residents. 

We determined the most current ZCTA-based population estimates by using data from the 2010 Decennial US Census. ZCTAs are geographic units created by the US Census Bureau to aggregate census boundaries into ZIP code–like areas (4). We obtained ZIP code–level all-cause mortality data for the years 2008 through 2012, chosen to align most closely with the 2010 denominator data, from the Office of Health Status Monitoring, Hawai’i State Department of Health. We crosswalked ZIP code to ZCTA by using an established method (5). We parsed ZCTA-level data into 5-year age groupings (<1, 1–4, 5–9, . . . ≥85 y). We developed ZCTA-, county-, and state-level life expectancy estimates by using the Sub-County Assessment of Life Expectancy (SCALE) methodology (6), which uses an adjusted Chiang II methodology in the form of a validated tool from the South East Public Health Observatory (7). We suppressed any life expectancy estimate with a standard error of 2 years or more (n = 21). Because of a negligible population in Kalawao County (N = 90), on Moloka`i, we grouped this county with Maui County. We used ArcMap 10.2 (Esri) to develop a ZCTA-based life expectancy map with quintile groupings.

## Main Findings

Hawai`i contains 94 ZCTAs, with populations varying from 0 to 72,289 residents (median, 4,528). From 2008 through 2012, an annual average of 9,553 deaths occurred among Hawai`i residents, ranging from 0 to 541 deaths per ZCTA (median, 27). The map shows 73 (78%) ZCTAs across Hawai`i with reportable life expectancy estimates. Honolulu County had the highest proportion of reportable life expectancy estimates (31 of 35 ZCTAs), followed by Maui (12 of 15 ZCTAs), Hawai`i (20 of 28 ZCTAs), and Kaua`i (10 of 16 ZCTAs) counties.

Overall, the state average life expectancy was 82.3 years (95% confidence interval [CI], 82.1–82.4 y), similar to previous estimates (9). All 4 counties had comparable life expectancy estimates, and each county estimate had a range of fewer than 2 years: Honolulu County had the highest life expectancy (82.6 y; 95% CI, 82.4–82.7 y), followed by Maui (82.3 y; 95% CI, 81.8–82.7 y), Kaua`i (81.9 y; 95% CI, 81.3–82.5 y), and Hawai`i (80.9 y; 95% CI, 80.5–81.3) counties. Life expectancy varied by ZCTA, ranging from 72.9 years (95% CI, 69.4–76.3 y) to 87.3 years (95% CI, 86.3–88.3 y), a 14.4-year difference. Honolulu County had the ZCTA with the highest life expectancy in the state (87.3 y) and the most ZCTAs in the top quintile (83.5–87.3 y); however, it also had the widest range in subcounty life expectancy (12.4 y). The ZCTA life expectancy range in Maui County was 11.6 years, and the island of Moloka`i had the ZCTA with the lowest life expectancy in the state (72.9 y). Hawai`i County had the most ZCTAs (n = 6) in the lowest quintile (72.9–79.9 y) and a life expectancy range of 8.9 years. Finally, Kaua`i County had the smallest subcounty life expectancy range (8.3 y).

## Action

Subcounty life expectancy estimates are critical to understanding whether life expectancy varies across Hawai`i. Our map revealed that county-level life expectancy estimates, which show little variation among counties, mask substantial subcounty differences. Therefore, county-level estimates are insufficient for understanding health disparities by geography. Among legislators, key stakeholders, and the public, visual tools such as maps enhance awareness of health disparities. Life expectancy is a cross-cutting health indicator: it cuts across diseases and identifies communities that require contributions across public health partners toward a common goal of alleviating the burden of all-cause mortality. Our study, however, has limitations: ZIP codes and ZCTAs can vary widely in population size and geographic area, and boundaries frequently change. Additionally, life expectancy is a measure of birth and death, not a full representation of population health.

Our map has several potential applications. For example, it can identify areas requiring targeted resources for reducing preventable causes of health disparities. The map could also be used to target health care providers in certain geographic areas for intensive quality improvement interventions for early detection and management of diseases. It could be incorporated into community needs assessments to support other subcounty health data. Finally, the map could be used to monitor progress toward reducing health disparities across the state. This map has been shared with partners in Hawai`i, including state agencies, large community organizations, and health plans.

## References

[1] Finch BK , Phuong Do D , Heron M , Bird C , Seeman T , Lurie N . Neighborhood effects on health: concentrated advantage and disadvantage. Health Place 2010;16(5):1058–60. 10.1016/j.healthplace.2010.05.009 20627796 PMC2918664

[2] Lewis K , Burd-Sharps S . American human development report: the measure of America 2013–2014. Measure of America of the Social Science Research Council. 2016. http://www.measureofamerica.org/wp-content/uploads/2013/06/MOA-III.pdf. Accessed June 20, 2018.

[3] Murray CJL , Michaud CM , McKenna MT , Marks JMUS . Patterns of mortality by county and race: 1965–1994. Cambridge (MA): Harvard Center for Population and Development Studies; 1998.

[4] US Census Bureau. Geographic terms and concepts – ZIP code tabulation areas. https://www.census.gov/geo/reference/gtc/gtc_zcta.html. Accessed June 22, 2018.

[5] Robert Graham Center. Uniform Data System mapper. https://www.graham-center.org/rgc/maps-data-tools/interactive/uds-mapper.html. Accessed January 11, 2018.

[6] Boothe VL , Fierro LA , Laurent A , Shih M . Sub-county life expectancy: a tool to improve community health and advance health equity. Prev Chronic Dis 2018;15:E11. 10.5888/pcd15.170187 29369759 PMC5798219

[7] Eayres D , Williams ES . Evaluation of methodologies for small area life expectancy estimation. J Epidemiol Community Health 2004;58(3):243–9. 10.1136/jech.2003.009654 14966240 PMC1732705

[8] Hawaii State Data Center. Research and Economic Analysis Division, Department of Business, Economic Development and Tourism, State of Hawaii. Urban and rural areas in the state of hawaii, by county: 2010. 2013. http://files.hawaii.gov/dbedt/census/Census_2010/Other/2010urban_rural_report.pdf

[9] Wu Y , Braun K , Onaka AT , Horiuchi BY , Tottori CJ , Wilkens L . Life expectancies in Hawai’i: a multi-ethnic analysis of 2010 life tables. Hawaii J Med Public Health 2017;76(1):9–14. 28090398 PMC5226016

